# Wide-Awake Tenolysis for Tendon Entrapment in a Distal Radial Epiphyseal Separation (Volar Displacement Type): A Case Report

**DOI:** 10.7759/cureus.63837

**Published:** 2024-07-04

**Authors:** Yusuke Mori, Ken Yamamoto, Naoya Kubota, Koichi Yano

**Affiliations:** 1 Orthopaedics, Ishikiri Seiki Hospital, Higashiōsaka, JPN; 2 Orthopaedics, Tsuji Surgical Rehabilitation Hospital, Osaka, JPN; 3 Orthopaedic Surgery, Seikeikai Hospital, Sakai, JPN

**Keywords:** extensor digitorum communis tendon, volar displacement type, distal radial epiphyseal separation, walant technique, tendon entrapment

## Abstract

The patient was a 13-year-old male who fell while riding a bicycle and was initially diagnosed with a distal radial epiphyseal separation (volar displacement type) that was conservatively managed. Four months post-injury, he complained of limited movement in his left index finger and was referred to our hospital. Upon examination, the patient also complained of limited movement of the left index finger in wrist flexion. The wrist range of motion was 50° of volar flexion, 50° of dorsiflexion, 90° of pronation, and 90° of supination with the fingers extended. The X-ray revealed a radiolucent area in the distal radius. Ultrasound, computed tomography, and magnetic resonance imaging scans demonstrated entrapment of the extensor tendon within the medullary cavity of the radius. Five months post-injury, surgery was performed using the wide-awake local anesthesia no-tourniquet (WALANT) technique. A dorsal wrist approach was utilized, and the extensor digitorum communis tendon was found to be trapped within the medullary cavity of the radius. The tendon was released using an air drill, and sufficient improvement in the left index finger flexion was confirmed with active movement before concluding the surgery. At the 11-month postoperative follow-up, the patient showed excellent outcomes with a wrist range of motion of 75° of volar flexion, 85° of dorsiflexion, 90° of pronation, and 90° of supination. Tendon entrapment of the extensor tendons has been reported as a long-standing complication associated with distal radius fractures, particularly with volar displacement types. A benefit of the WALANT technique is the ability to communicate with the patient during surgery, allowing for active movements of the fingers and wrist. This is particularly useful in tendon surgeries for determining tendon tension. We report a case of successful tenolysis surgery using the WALANT technique for a patient with a conservatively managed distal radial epiphyseal separation (volar displacement type), who experienced a limited flexion of the index finger due to tendon entrapment.

## Introduction

Tendon entrapment associated with distal radius fractures is a relatively rare complication but has been frequently reported in the literature. Most of these fractures are of the volar displacement type, which is more unstable than that of the dorsal displacement type; therefore, such fractures require careful attention [1].

The wide-awake local anesthesia no tourniquet (WALANT) technique, which provides a bloodless field using local anesthesia with epinephrine without a tourniquet, is gaining attention as a surgical method that challenges the conventional use of tourniquets in hand surgery. The ability to perform the surgery while allowing active movements of the fingers and wrist is a benefit of this technique [2].

We report a case of a patient with a conservatively managed distal radial epiphyseal separation (volar displacement type), who developed a limitation in the flexion of the left index finger due to extensor tendon entrapment.

## Case presentation

**Figure 1:**
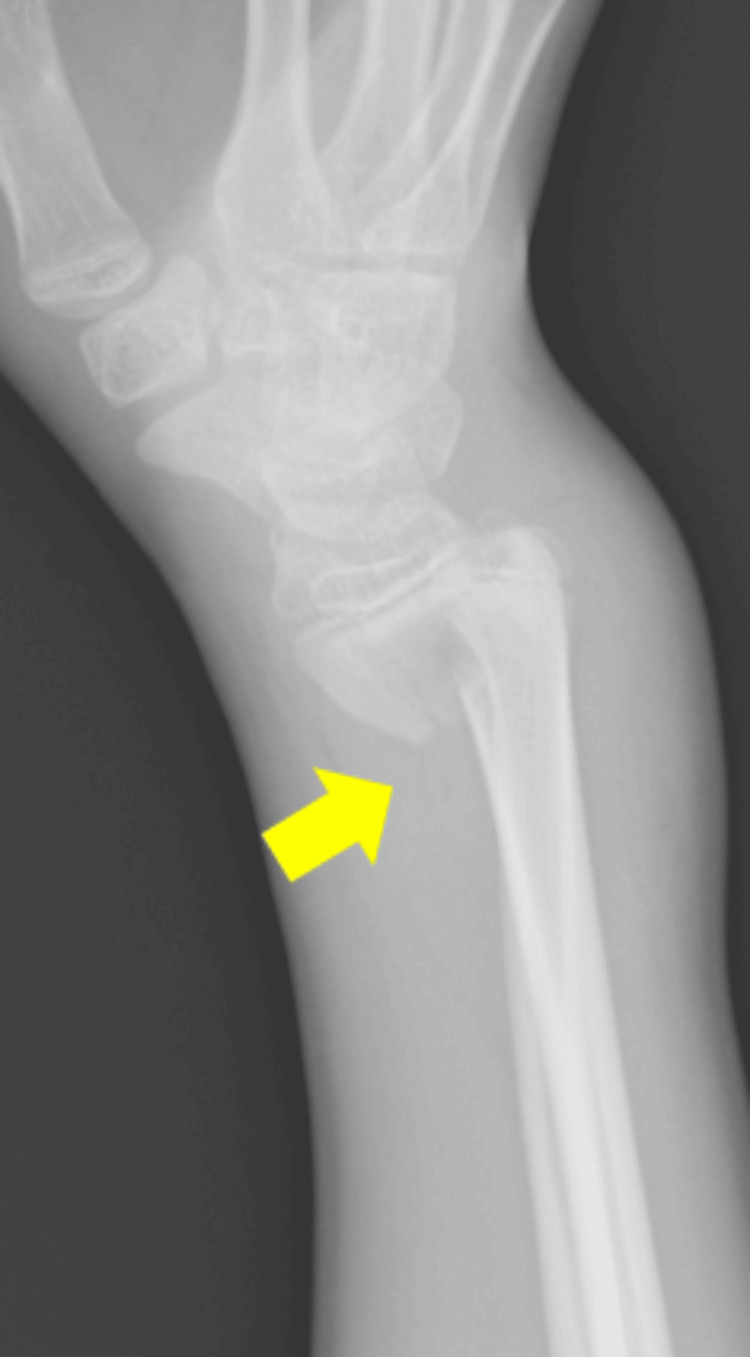
X-ray at the time of injury (yellow arrow)

**Figure 2:**
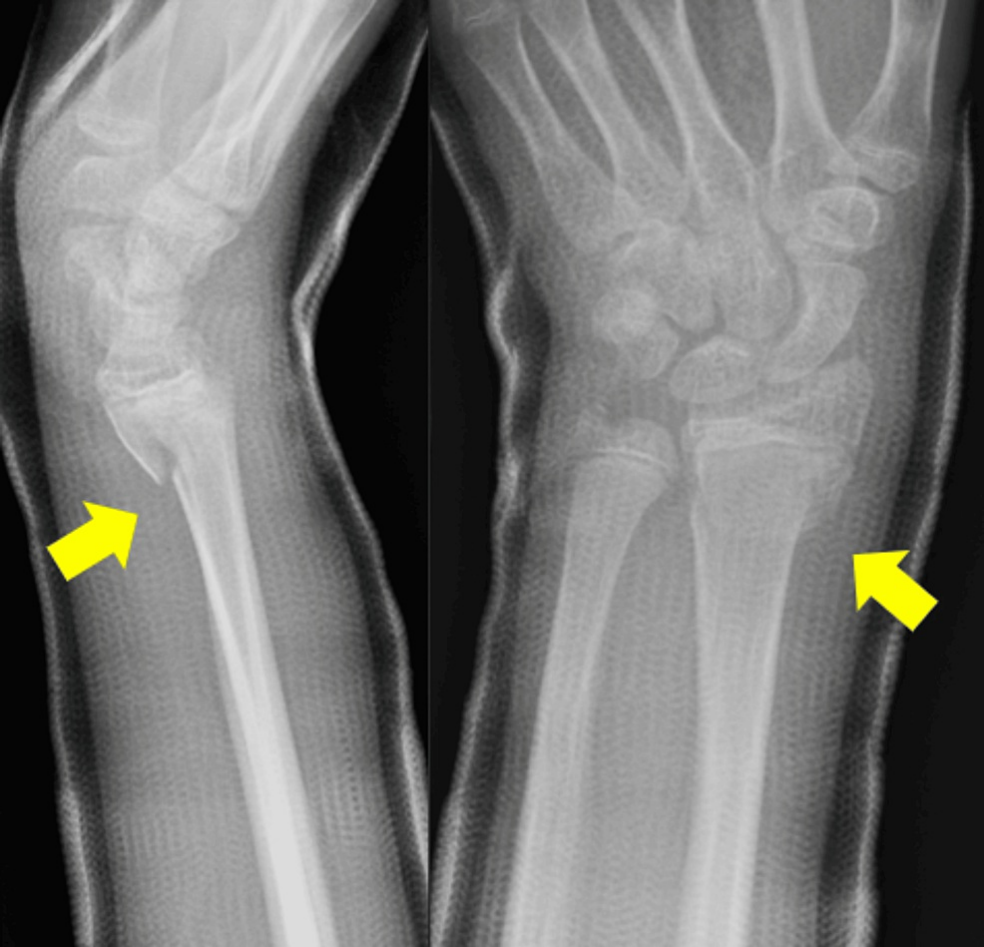
X-rays after manual reduction and immobilization (yellow arrow)

**Figure 3:**
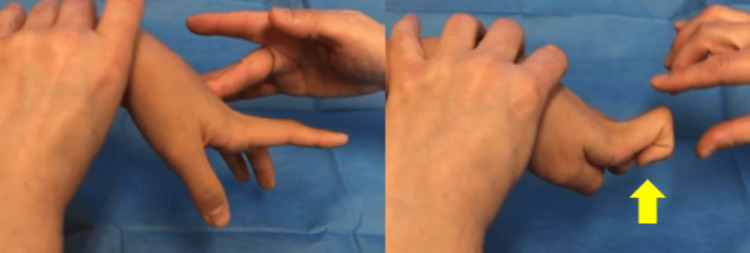
Limited flexion of the index finger in wrist flexion (yellow arrow)

**Figure 4:**
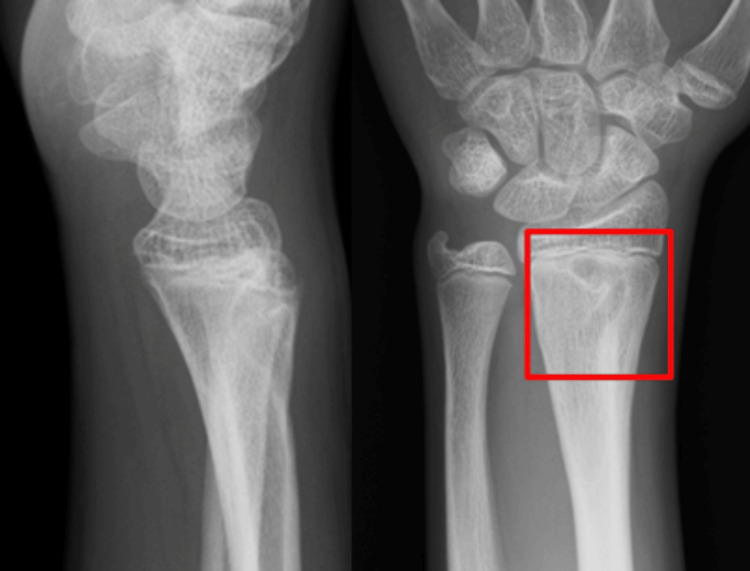
X-ray showing a radiolucent area in the distal radius (red box)

**Figure 5:**
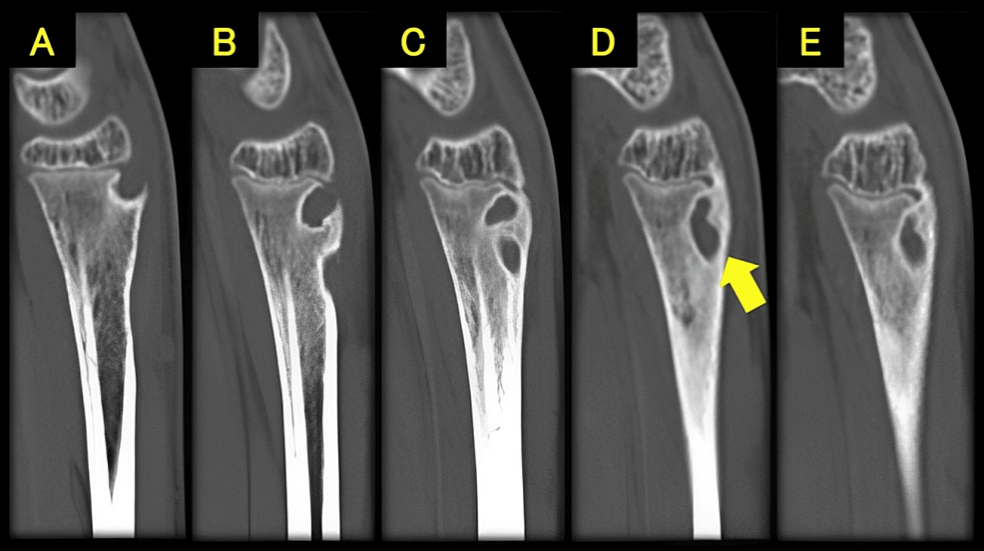
Computed tomography scans (sagittal view) showing a radiolucent area in the distal radius (yellow arrow) Sagittal view: from A to E, ulnar to radial

**Figure 6:**
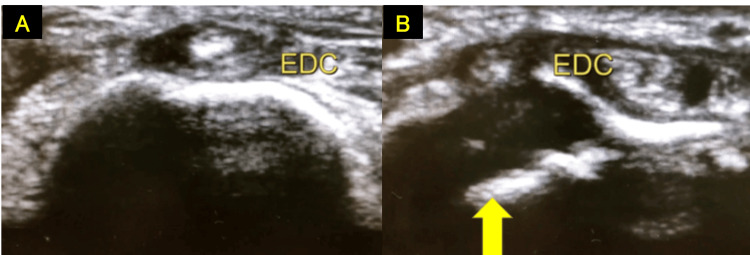
Ultrasound of the wrist showing EDC entrapment within the medullary cavity of the radius A: Normal. B: EDC entrapment within the medullary cavity of the radius (yellow arrow). EDC, extensor digitorum communis

**Figure 7:**
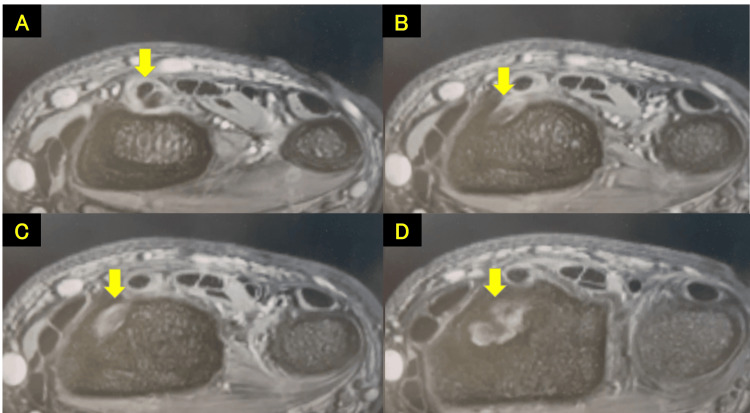
Magnetic resonance imaging scans showing EDC entrapment within the medullary cavity of the radius (yellow arrow) From A to D, cranial to caudal. EDC, extensor digitorum communis

A 13-year-old boy fell while riding a bicycle and was initially diagnosed at another hospital with a Salter-Harris type II distal radial epiphyseal separation with volar displacement (Figure 1). The fracture was manually reduced and immobilized with a cast for four weeks (Figure 2). Four months post-injury, he complained of limited movement in his left index finger and was referred to our hospital. On examination, finger movements were normal in wrist dorsiflexion. However, in wrist volar flexion, the index finger could not fully flex, showing hyperextension of the metacarpophalangeal joint (Figure 3, Video 1). The wrist range of motion was 50° of volar flexion, 50° of dorsiflexion, 90° of pronation, and 90° of supination. The Visual Analog Scale (VAS) and Quick Disabilities of the Arm, Shoulder, and Hand (Q-DASH) scores were 0/10 and 18, respectively. X-ray and computed tomography scans revealed a radiolucent area in the distal radius (Figures 4, 5). Ultrasound and magnetic resonance imaging scans demonstrated that the extensor digitorum communis (EDC) tendon of the index finger (EDC2) was trapped within the medullary cavity of the radius, which led to the decision to perform tenolysis surgery (Figures 6, 7).

**Video 1:** Limited flexion of the index finger in wrist flexion

**Figure 8:**
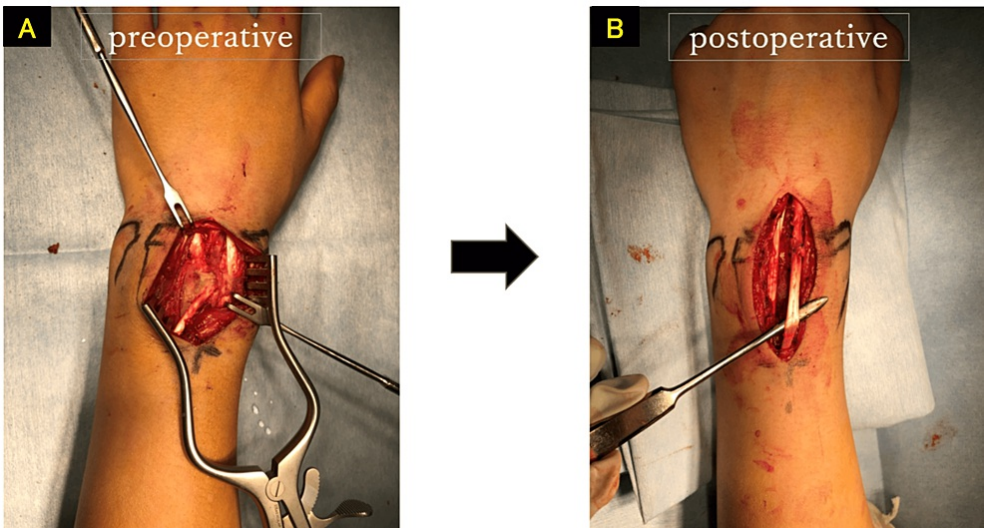
Extensor tendon tenolysis performed (four months post-injury) A: preoperative. B: postoperative.

**Figure 9:**
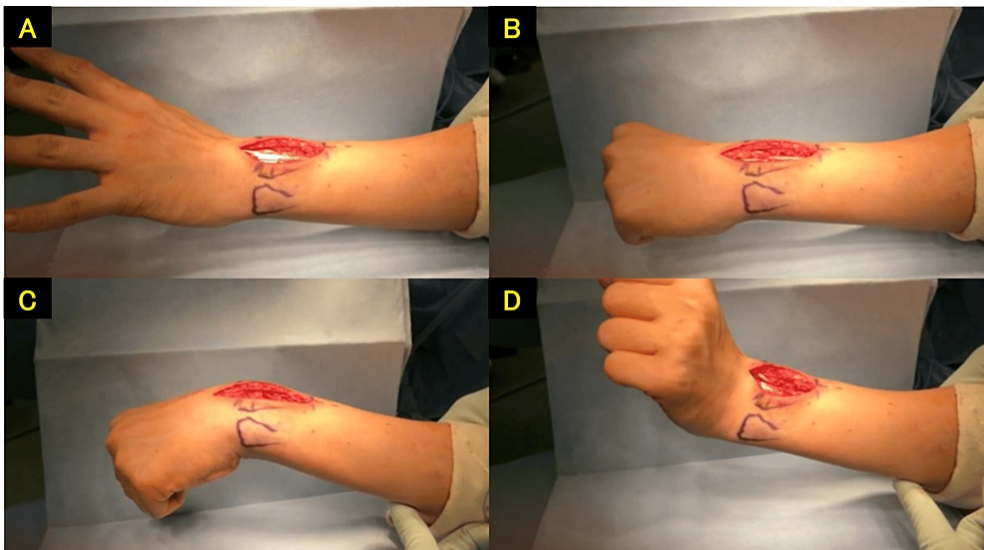
Surgery performed using the WALANT technique, confirming improvement in the index finger flexion with intraoperative active movement A: finger extension. B: finger flexion. C: wrist dorsiflexion. D: wrist volar flexion.

**Figure 10:**
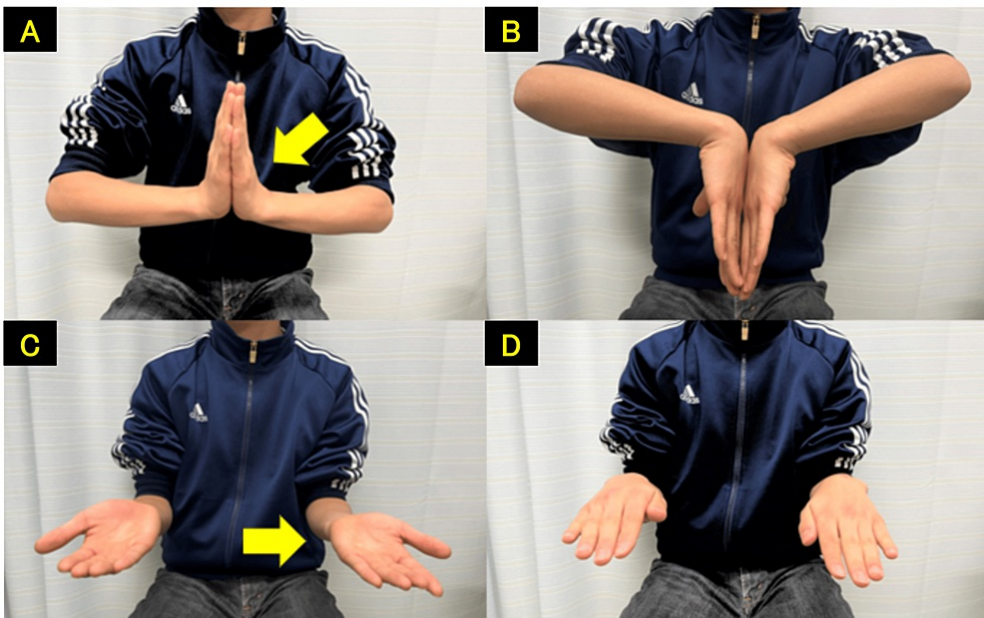
Wrist range of motion at 11 months post-operation A: 75°of volar flexion. B: 85°of dorsiflexion. C: 90°of supination. D: 90°of pronation (yellow arrow: injured side)

**Figure 11:**
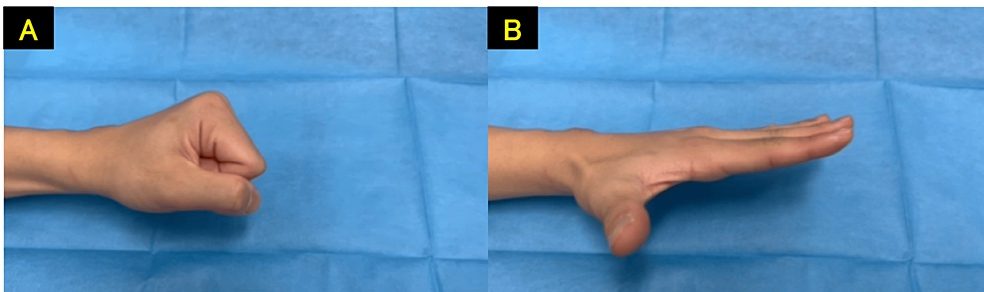
No recurrence of the limited index finger flexion at 11 months post-operation A: finger flexion. B: finger extension.

The surgery was performed using the WALANT technique. The local anesthetic used was a mixture of 20 ml of ropivacaine and 10 ml of lidocaine with epinephrine. A dorsal wrist approach was used to release the extensor retinaculum and expose the fracture site. EDC2 was found to be trapped within the medullary cavity of the radius, approximately 3-5 cm proximal to the joint surface. An air drill was used to excavate the bone surrounding the extensor tendon and resolve the entrapment. Sufficient improvement in the left index finger flexion was confirmed with active movement, and the surgery was concluded (Figures 8, 9). Eleven months post-operation, the wrist range of motion was 75° of volar flexion, 85° of dorsiflexion, 90° of pronation, and 90° of supination (Figures 10, 11). Grip strength was 25 kg on the right and 28.9 kg on the left, and the VAS and Q-DASH scores were 0/10 and 7, respectively. There was no evidence of growth disturbance in the distal radius, and the postoperative course was favorable.

## Discussion

Tendon entrapment associated with distal radius fractures was first reported by Hunt in 1969, who identified extensor tendon entrapment in volar displacement-type distal radius fractures [3]. The most commonly entrapped tendons are the extensor pollicis longus [4-6], extensor indicis proprius [6], and the EDC [4,6]. Okazaki et al. reported tendon entrapment in eight out of 633 acute distal radius fractures (1.3%) and in one out of 68 cases (1.5%) of malunion. Of these, five out of nine cases (55.6%) involved distal radial epiphyseal separations in children, and eight out of nine cases (88.9%) were of the volar displacement type [6].

In addition, Thomas, Uchida, El-Kazzi, and Lee reported extensor tendon entrapment in volar displacement-type distal radial epiphyseal separations in children, with many cases specifically involving Salter-Harris type II fractures [7-10].

**Figure 12:**
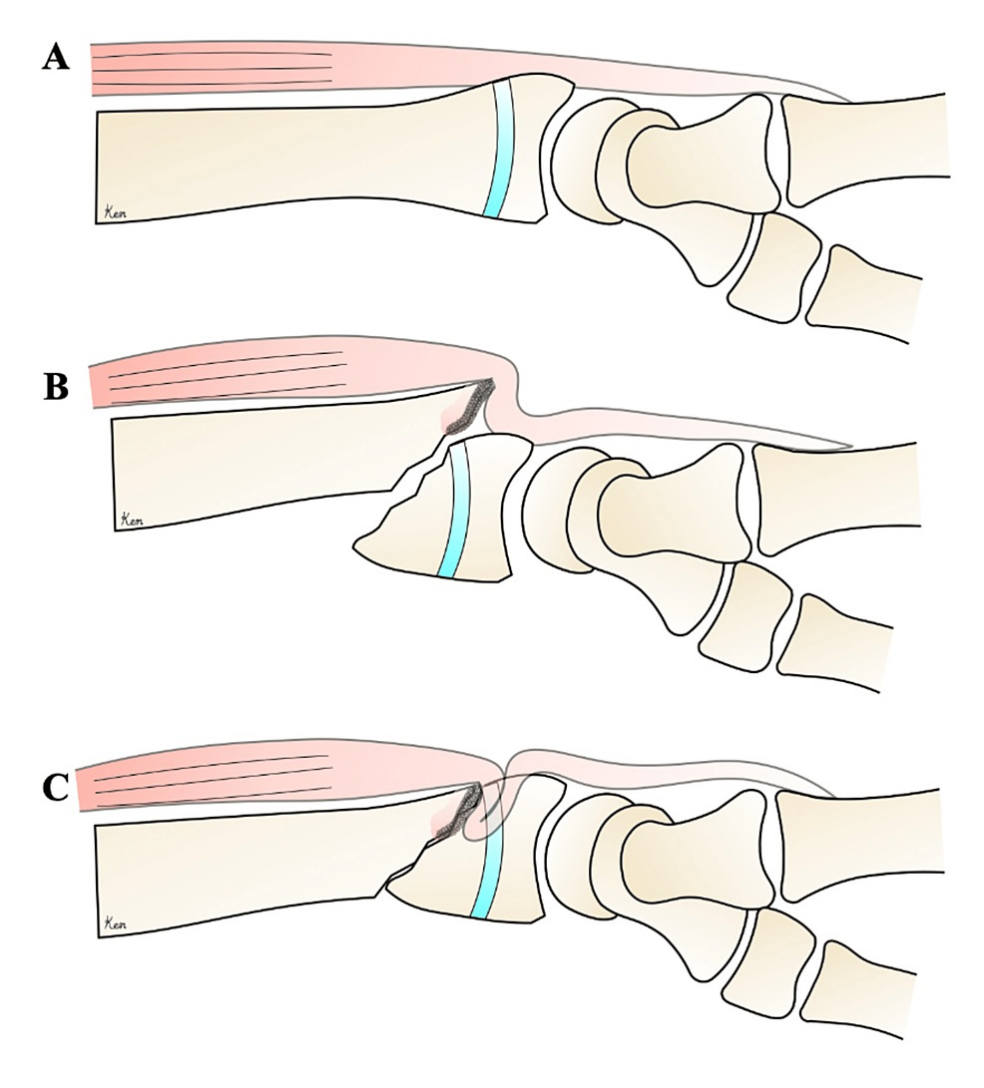
A. Normal. B. At the time of injury. C. Tendon entrapment after reduction The image was created by Ken Yamamoto.

This case involved a distal radial epiphyseal separation with significant volar displacement. We considered that the sharp distal dorsal radial fragment impinged on the extensor tendon (EDC2) during manual reduction (Figure 12). Based on this, we suggest that careful attention should be paid during the reduction of distal radial epiphyseal separations with volar displacement.

The WALANT technique represents a significant recent advancement in hand surgery, providing a bloodless field using local anesthesia with epinephrine without a tourniquet [2]. This method is safer for patients with a history of dialysis or severe pulmonary disease. Furthermore, WALANT allows for intraoperative communication with the patient, enabling active movements of the fingers and wrist, which is beneficial for determining tendon tension during tendon surgeries.

There are many studies reporting the benefits of the WALANT technique. Zukawa et al. reported a study on tendon reconstruction using the WALANT technique for chronic flexor tendon ruptures. The results showed that measuring total active motion (TAM) and adjusting tendon tension intraoperatively contributed to improved functional outcomes, making WALANT a superior choice for tendon surgeries [11].

Peter et al. conducted a prospective study on 100 hand surgery cases using the WALANT technique and found that 94% of patients preferred WALANT for similar future surgeries. In addition, WALANT was significantly more cost-effective, suggesting its potential benefits for patients [12].

In this case, considering the chronic nature of the injury four months post-trauma, the WALANT technique was chosen to facilitate the intraoperative determination of tendon tension. Tenolysis, tendon graft, and tendon transfer are surgical options for tendon entrapment. In this case, tenolysis alone was sufficient as there was no intraoperative evidence of tendon rupture, and full grip was achieved with active movement using the WALANT technique.

## Conclusions

We report a case of a distal radial epiphyseal separation (volar displacement type) that was initially treated without surgery, resulting in limited flexion of the left index finger due to extensor tendon entrapment. The patient was successfully treated with tenolysis surgery using the WALANT technique. The WALANT technique allowed for intraoperative communication with the patient, facilitating the surgical process and contributing to symptom improvement in this case.
